# Annotations

**Published:** 1904-11-12

**Authors:** 


### Annotations.

### The Parliamentary Representation of the Universities of Glasgow and Aberdeen.

The announcement of the intention of Dr. Jas. A. Campbell not to seek re-election at the close of the present Parliament has been followed by a series of manœuvres which have produced a somewhat curious position. The Conservative and Unionist Associations, the headquarters of which are naturally at the Universities in question, decided to nominate Sir Henry Craik, K.C.B., for the vacancy, and to this proposal they appear to have received the cordial assent of their representatives in London. Hence Sir Henry Craik was formally announced as the candidate approved by those who would seem to have the right to speak for the Conservative and Unionist interests in the constituency, and in view of his long and intimate acquaintance with the various problems of Scottish education, no one, whatever his party, could be surprised at the selection. Granted the legitimacy of University representation, an educational authority of the first rank would appear, political considerations apart, to be a peculiarly suitable representative of an academic constituency, and a general agreement and unanimous election seemed, therefore, not improbable. But opposition has suddenly sprung from an unexpected and somewhat mysterious quarter. While the Liberal Party has taken no active steps to oppose Sir Henry Craik's candidature, a rival holding the same political faith has come into the field in the person of Dr. W. R. Smith. This is, so far, comparatively plain sailing, but the curious thing is that the last-named gentleman is advanced as the nominee of a second association which claims to represent Unionist interests in the constituency. This association, it is said, was formed in London some few years ago, but by what right it placed itself in competition with the older official organisations does not appear. The practical position, therefore, is that the constituency is confronted by two candidates, each claiming to be the approved nominee of the Unionist Party, and thus the case would seem to be one for a careful scrutiny of credentials. An official and an independent candidate is, of course, not an infrequent experience in electoral contests. But here are two candidates boasting one and the same formal authority —and yet there cannot be two Simon Pures.

### Women Inebriates.

The reports which have been issued at various times from institutions occupied with the attempt to reform women inebriates have not been cheerful reading and the latest of the series, that from the London County Council Home at Farmfield, forms no exception to the rule. Thus of 22 patients licensed during the year no less than 11 relapsed and were brought back to the institution; and of the same number discharged eight have relapsed and only four are known to be doing well. No doubt the benefits of a return to sobriety in any individual case are beyond expression in the prosaic terms of pounds, shillings, and pence, but it has to be remembered, in justice to the ratepayer, that this gain is attained at considerable public expense. The authorities at Farmfield complain of the unsatisfactory nature of the majority of cases committed to their care and appear to despair of improved results until "a better type of woman" is sent to them. That, in plain terms, would appear to mean that there are a number of women inebriates who are beyond redemption by any known method of reformatory effort and discipline. But this is not all. For, according to the report in question, these hopeless cases exercise a prejudicial influence on those of a more promising order. This consideration renders the problem of dealing with such women a particularly urgent one. The public conscience will hardly approve of a proposal to turn them out on the streets, and still less of the "short way with degenerates" advocated in certain quarters. A more reasonable suggestion is to confine such persons in institutions more or less resembling prisons. The position, in short, calls for robust common sense and not for a flabby sentimentality. It is necessary to recognise the fact that in certain of these cases efforts at reform are merely waste of time and money, and that it is unfair to the ratepayer to conduct numberless experiments at his charge. To cope successfully with inebriety, as is the case also with many other diseases, demands preventive rather than curative measures, and in this direction there is much opportunity for both State and Municipal effort.

### The Sale of Infectious Milk.

The facts brought before the jury during the action for damages recently brought against a large dairy company for supplying infective milk are sufficiently important to call for notice. The suspected milk had been obtained by the dairy company from a farm in Berkshire which, like the other farms supplying them, was subject to periodical inspection by the local medical officer of health, and was supposed to be conducted under regulations calculated to afford safety against infection. It appeared, nevertheless, that there was a source of polluted water upon the premises, which was known to have occasioned illness to those who drank from it, and the use of this water for other purposes than swilling the floors was forbidden. Water for washing the dairy utensils was brought every day in a tank cart from a spring two and a half miles away; but it was shown that while the cart held only 180 gallons, and while the daily requirement of pure water approached 400 gallons, it was not every day that a second journey was made in order to procure it. The evidence that milk was the source of the illness was sufficient to satisfy the jury; and Mr. Justice Grantham put it almost as a matter of common sense that the class of people employed as dairy workers were not likely to be deterred by sanitary considerations from surreptitiously using, when a permitted water supply had failed, that which was readily accessible. It appeared that the well on the farm had been officially condemned by the medical officer of health, but that, in the words of his evidence, "they had not yet got a proper supply." It would therefore seem, *prima facie*, as if the responsibility for the deaths occasioned rested primarily upon the sanitary (?) authority of the district; and it is much to be regretted that the law makes no provision for bringing home such responsibility to the guilty persons.

